# Involvement of the long noncoding RNA *H19* in osteogenic differentiation and bone regeneration

**DOI:** 10.1186/s13287-021-02149-4

**Published:** 2021-01-21

**Authors:** Zimo Zhou, Mohammad Showkat Hossain, Da Liu

**Affiliations:** Department of Orthopedics, Shengjing Hospital of China Medical University, Shenyang, 110004 Liaoning China

**Keywords:** Long noncoding RNA, *H19*, Osteogenic differentiation, Bone regeneration

## Abstract

Osteogenic differentiation and bone regeneration are complex processes involving multiple genes and multiple steps. In this review, we summarize the effects of the long noncoding RNA (lncRNA) *H19* on osteogenic differentiation.

Osteogenic differentiation includes matrix secretion and calcium mineralization as hallmarks of osteoblast differentiation and the absorption of calcium and phosphorus as hallmarks of osteoclast differentiation. Mesenchymal stem cells (MSCs) form osteoprogenitor cells, pre-osteoblasts, mature osteoblasts, and osteocytes through induction and differentiation. lncRNAs regulate the expression of coding genes and play essential roles in osteogenic differentiation and bone regeneration. The lncRNA *H19* is known to have vital roles in osteogenic induction.

This review highlights the role of *H19* as a novel target for osteogenic differentiation and the promotion of bone regeneration.

## Introduction

Bone regeneration is a complex process involving the synergistic effects of mesenchymal stem cell (MSC)-derived osteoblasts and hematopoietic stem cell-derived osteoclasts. After fracture or the onset of osteoporosis and other diseases, the damaged bone releases cytokines. These cytokines induce osteoblastic matrix secretion and calcium mineralization. MSCs gradually differentiate into bone progenitor cells, pre-osteoblasts, and osteoblasts. Then, osteoblasts begin to synthesize and secrete matrix, repair the tissue microenvironment, and induce bone regeneration. Moreover, with the differentiation of osteoclasts, the organic and inorganic compounds released by the damaged bone are absorbed. Ca^2+^, (PO_4_)^3−^, and other degradation products enter the blood circulation. These processes work effectively through complex multigene processes, with multistep regulation.

**Fig. 1:**
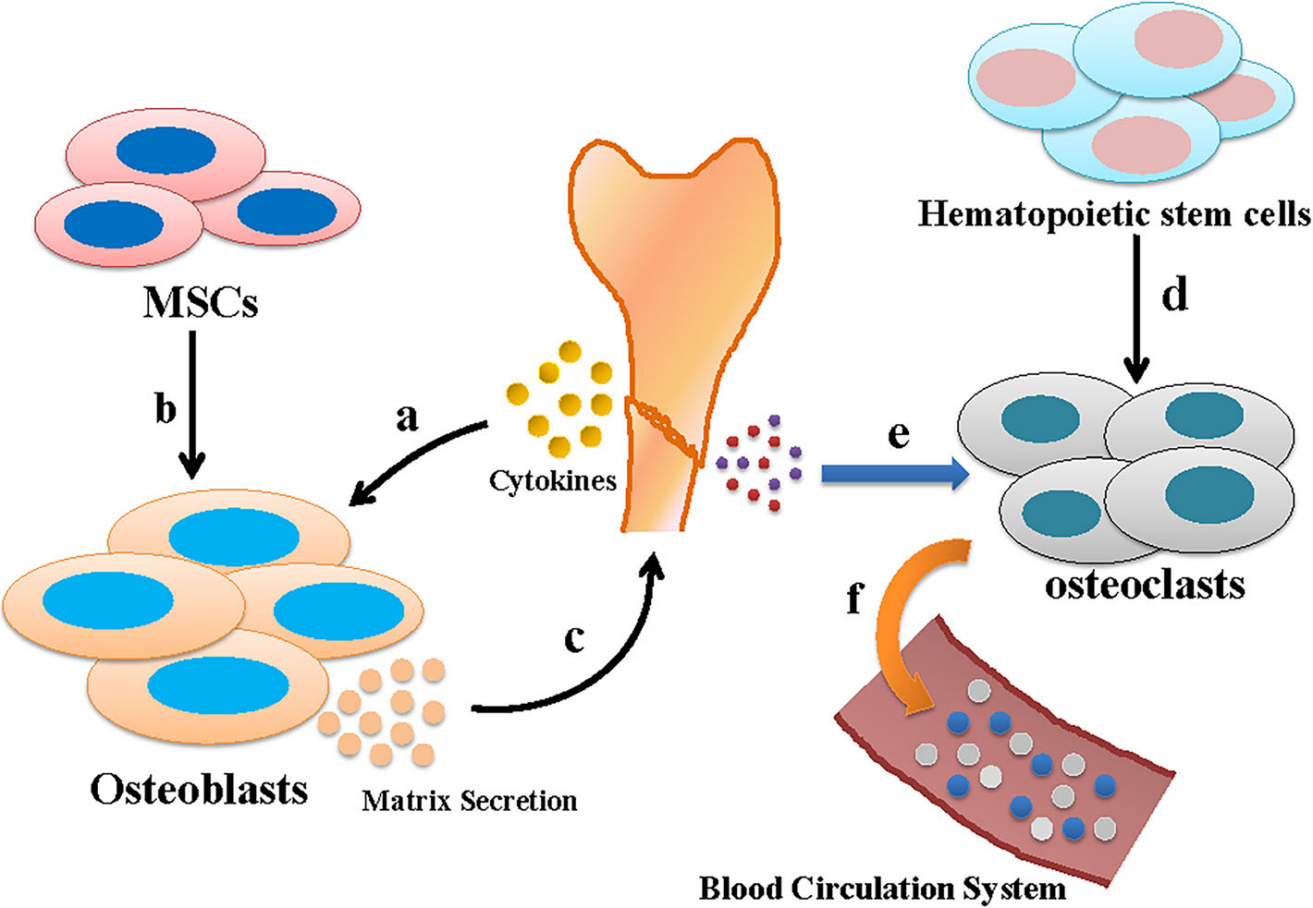
Synergistic effects of MSC-derived osteoblasts and hematopoietic stem cell-derived osteoclasts. (**a**) Damaged bone will release cytokines to induce osteoblastic matrix secretion and calcium mineralization. (**b**) MSCs differentiate into osteoblasts. (**c**) Osteoblasts start to synthesize and secrete matrix, repair the tissue microenvironment, and induce bone regeneration. (**d**) Hematopoietic stem cells differentiate into osteoclasts. (**e**) Osteoclasts absorb organic and inorganic compounds released by damaged bone. (**f**) Ca^2+^, (PO_4_)^3−^, and other degradation products enter the blood circulation system

MSCs are stem cells with multipotent differentiation capacity. Many studies have demonstrated that MSCs play crucial roles in maintaining and repairing various connective tissues, including cartilage, muscle tissue, bone, and adipose tissues [1]. As an essential process in bone regeneration and cell repair, the osteogenic differentiation potential of MSCs is induced by the extracellular microenvironment. Indeed, mechanical and molecular signals regulate osteogenic differentiation at the transcriptional and post-transcriptional levels [2] (Fig. 1).

In prior studies, the roles of protein-coding genes and noncoding microRNAs in osteogenic differentiation have been extensively studied. However, long noncoding RNAs (lncRNAs), which account for a large proportion of the genome sequence, have not been sufficiently studied. With the recent development of high-throughput RNA sequencing (RNA-seq) and other technologies, lncRNAs, previously regarded as transcriptional noise, have been shown to have positive roles in regulating nuclear chromatin structure and gene expression. Zuo et al. [3] first reported the relationship between lncRNAs and bone generation in 2013. In response to bone morphogenetic protein-2 (BMP-2), lncRNA expression profiles are significantly altered in C3H10T1/2 cells, demonstrating a correlation between lncRNAs and osteoblast differentiation. Moreover, researchers have identified 116 differentially expressed lncRNAs, facilitating further studies of these sequences in osteogenesis.

In this review, we summarize the effects of the lncRNA *H19* on osteogenic differentiation. We also discuss the roles of other lncRNAs associated with this process and highlight the potential applications of this information regarding the understanding and management of bone-related diseases.

## Structure and function of lncRNAs

lncRNAs, as by-products of RNA polymerase II transcription, belong to a family of noncoding RNAs (ncRNAs) with lengths of 200–100,000 nt. These molecules have little or no protein-coding potential [4, 5]. Functionally, lncRNAs act as regulatory ncRNAs and include microRNAs (miRNAs), small interfering RNAs (siRNAs), and Piwi-interacting RNAs [6]. Compared with miRNAs, lncRNAs show lower expression levels and exhibit relatively low homology among species. However, promoters and exons are conservative to some extent, indicating that the functions of lncRNAs are relatively conserved [7]. Many lncRNAs contain conserved secondary structures and exhibit alternative splicing and subcellular localization. In addition, many lncRNAs show specific expression during various stages of tissue development.

lncRNAs can be divided into five types: sense, antisense, bidirectional, intronic, and intergenic; the functions of these lncRNAs differ to some extent [2, 8]. In general, lncRNAs have no coding potential; however, Matsumoto et al. [9] showed that a small polypeptide encoded by the lncRNA *LINC00961* could inhibit the amino acid-induced activation of skeletal muscle mammalian target of rapamycin complex 1 in SPAR-polypeptide-specific-knockout mice, demonstrating that lncRNAs could encode short peptides under exceptional circumstances.

## Regulatory mechanisms of lncRNAs in osteogenic differentiation

The regulatory mechanisms of lncRNAs are highly complex. The mechanisms of action of lncRNAs can be summarized into four levels: epigenetic, transcriptional, post-transcriptional, and other regulatory mechanisms. In osteogenic differentiation, lncRNAs show three general functional roles, as follows: (1) they mediate epigenetic modification to regulate osteogenic differentiation; (2) they regulate osteogenic differentiation through the modulation of signaling pathways; and (3) they regulate osteogenic differentiation by serving as miRNA sponges or precursor structures.

### Roles of lncRNAs in mediating epigenetic modifications to regulate osteogenic differentiation

Epigenetics refers to heritable genetic phenotypes and gene expression changes through DNA methylation, histone modification, and chromatin remodeling without changes in the DNA sequences. DNA methylation can directly regulate the expression of Runt-related transcription factor 2 (Runx2) and osterix (Osx), which affect bone formation [10]. Kino et al. [11] showed that during osteogenic differentiation, the lncRNA *Gas5* could bind to the glucocorticoid receptor gene binding domain as bait and inhibit receptor function. As negative regulators of bone formation, glucocorticoids cannot bind to glucocorticoid receptors.

### Roles of lncRNAs in regulating osteogenic differentiation through modulation of signaling pathways

A series of regulatory factors and cells are involved in osteogenesis and osteogenic differentiation. These regulators play important roles by activating or inhibiting relevant signaling pathways. The Wnt/β-catenin, mitogen-activated protein kinase (MAPK), and BMP/Smad pathways have been extensively studied [12–14]. The core transcription factor of osteogenic differentiation, Runx2, can be modulated by BMPs, Wnt protein, estrogen, and glucocorticoids, resulting in alterations in the phosphorylation or expression of downstream elements, such as β-catenin and Smads [15, 16]. SiRNAs have inhibitory effects on the activity of the lncRNA *AK045490*, which can promote osteoblastic differentiation in the context of osteoporosis. Moreover, experimental results have shown that *AK045490* downregulates T cell factor 1 (TCF1), lymphoid enhancer-binding factor 1 (LEF1), and Runx2 by inhibiting the nuclear translocation of β-catenin, blocking the β-catenin/TCF1/Runx2 signaling pathway, and ultimately suppressing the differentiation and bone formation of osteoblasts [15]. Additionally, HOX transcript antisense RNA (*HOTAIR*) can directly reduce Wnt inhibitory factor 1 (WIF-1) expression by promoting histone H3K27 methylation in the promoter region, thereby regulating the Wnt/β-catenin signaling pathway and activating matrix metalloproteinase-13 (MMP-13) expression in chondrocytes to block cartilage damage [17–19]. Inflammatory signals play essential roles in inducing osteogenic differentiation through the matrix microenvironment. In the osteogenic differentiation of human MSCs, the lncRNA differentiation antagonizing non-protein-coding RNA (*DANCR*) induces the expression of interleukin (IL)-6 and tumor necrosis factor-α (TNF-α) in mononuclear cells, thereby enhancing the osteoclastic activity of bone resorption [20]. IL-1β can also activate osteogenic differentiation via upregulation of the extracellular signal-regulated kinase (ERK) 1/2 signaling pathway. However, IL-1β eventually inhibits osteoblast differentiation via the strong activation of p38 signaling. Matrix stiffness can also regulate osteogenic differentiation by modulating the MAPK pathway [21, 22]. However, no reports have described the modulation of osteogenic differentiation through lncRNA-dependent inflammatory signals.

### Roles of lncRNAs as miRNA sponges or precursors to regulate osteogenic differentiation

miRNAs, which cause translational repression or degradation of target mRNAs, regulate the expression of genes involved in the osteogenic differentiation of MSCs. For example, *miR-138* inhibits osteoblast differentiation in bone marrow mesenchymal stem cells (BMSCs) and phosphorylation of focal adhesion kinase (FAK), ERK1/2, and Runx2. Moreover, *miR-138*-dependent downregulation of Runx2 is also essential for the platelet-derived growth factor (PDGF)-mediated inhibition of BMSC osteogenic differentiation [23], and *miR-705*, *miR-124*, *miR-204*, *miR-30a*, and *miR-705* regulate the balance between lipid formation and osteogenic differentiation in BMSCs by modulating Runx2 and Osx expression [24–28]. Studies have shown that lncRNAs can competitively associate to limited miRNA-specific sites and regulate miRNA levels. The lncRNA *KCNQ1OT1* interacts directly with *miR-214* to form an miRNA sponge during the regulation of BMSC osteogenic differentiation, and *miR-214* can bind to the 3′-untranslated region (UTR) of BMP-2 to inhibit the expression of this protein [29, 30].

Notably, some miRNAs can be transcribed from genomic regions of lncRNA gene sequences. As miRNA precursors, these molecules regulate downstream targets after being cleaved [31]. Additionally, lncRNAs can also facilitate the cleavage of pri-miRNAs, modulate the production of mature miRNAs, and play important regulatory role [32]. In one study using RNA-seq to elucidate the involvement of lncRNAs in the osteogenic differentiation of immortalized mesenchymal stem cells (iMSC#3), 32 new lncRNAs were screened out as miRNA precursors (including *miR-689*, *miR-640*, *miR-601*, and *miR-544*) [33]. Thus, further studies are expected to identify more functions of lncRNAs as miRNA sponges or precursors.

## Types and mechanisms of lncRNAs in osteogenic differentiation

Many lncRNAs have been shown to be involved in tumor growth, immune system diseases, and other diseases. For example, Luan et al. [34] knocked down the lncRNA *NPPA-AS1* in human normal cervical epithelial cells (H8 cells) and human cervical cancer cells (C33A, CaSki, HeLa, and SiHa cells) and showed that this lncRNA impaired cell proliferation and migration. Moreover, lncRNAs are known to participate in the progression of lung cancer, breast cancer, and cervical cancer [34–37]. Additionally, various lncRNAs can affect disease occurrence and outcomes through multiple molecular pathways. The various molecular mechanisms through which lncRNAs regulate osteogenic differentiation in disease are summarized in Table 1.

**Table 1:** The different types and roles of lncRNAs in osteogenic differentiation

Approved symbol	Gene locus	Change in expression	Target(s)	Stem cell types	Refs.
*DANCR*	4q12	Downregulated	*miR-1305*-Smad 4 axis EZH2, Runx2, OCN p38/MAPK pathway	PDLSCs hBMSCs	[36–41]
*HOTAIR*	12q13.13	Downregulated	Wnt/β-catenin pathway *miR-17-5p* Histone modification	BMSCs	[42–44]
*MALAT1*	11q13.1	Upregulated	Sponging for *miR-30* Sponging for *miR-214* Sponging for *miR-124* Sponging for *miR-34c* Sponging for *miR-204*	hBMSCs VICs	[27, 45–49]
*MEG3*	14q32.2	Upregulated Downregulated	*miRNA-543*/SMURF1/RUNX2 axis *miR-27a-3p*/IGF1 axis BMP4 signaling pathway	hDPSCs PDLSCs	[50–53]
*GAS5*	1q21	Upregulated Downregulated	*miRNA-498*/ RUNX2 axis *miR-26-5p*/PTEN axis *miR-135a-5p*/FOXO1 pathway GDF5 and p38/JNK signaling pathway	MSCs HASMCs	[1, 54–56]

### DANCR

The lncRNA *DANCR* was the first lncRNA shown to regulate progenitor differentiation [38]. The function of *DANCR* in chondrogenic differentiation of human synovium-derived MSCs and osteogenic differentiation of periodontal ligament stem cells (PDLSCs) has been reported [39, 40]. Additionally, Lin et al. [41] evaluated the expression of lncRNAs in hFOB1.19 human fetal osteoblastic cells and found that *DANCR* targets EZH2 and regulates the expression of Runx2 in osteogenic differentiation. During osteogenic differentiation, the canonical Wnt signaling pathway can be activated via *ANCR*-RNAi in PDLSCs during proliferation and osteogenic induction [42]. Moreover, *DANCR* has been shown to regulate the proliferation and osteogenic differentiation of human bone marrow-derived MSCs (PTA-1058 cells) via the p38/MAPK pathway [43].

### HOTAIR

*HOTAIR* is an lncRNA formed by *HOXC* gene transcription. *HOTAIR* can inhibit the activity of *HOX* and other target genes by chromatin remodeling [44]. In the osteogenic differentiation of BMSCs, *HOTAIR* mediates the Wnt/β-catenin pathway, and downregulation of *HOTAIR* results in the increased expression of Wnt/β-catenin pathway-related proteins [45]. Furthermore, during osteogenic differentiation and proliferation in nontraumatic osteonecrosis of the femoral head (ONFH), *HOTAIR* regulates osteogenic differentiation and proliferation by modulating the activity of the *miR-17-5p* and its target gene *Smad7* [46].

### Metastasis-associated lung adenocarcinoma transcript 1 (*MALAT1*)

*MALAT1* is a highly abundant and conserved imprinted gene. By investigating the function of *MALAT1* in calcific aortic valve disease, Xiao et al. [27] demonstrated that *MALAT1* could promote osteogenic differentiation. Additionally, Smad4 can be regulated by the *MALAT1*/*miR-204* sponge, promoting the osteogenic differentiation of calcific valves after osteogenic induction in human aortic valve interstitial cells. In another study, researchers found that *MALAT1* could regulate Osx expression by sponging *miR-143* to promote the osteogenic differentiation of human bone marrow-derived MSCs [47]. *MALAT1* can also promote osteogenic differentiation by sponging *miR-30*, *miR-214*, *miR-124*, and *miR-34c* [48–51].

### Maternally expressed gene 3 (*MEG3*)

The lncRNA *MEG3* is associated with various bone diseases, such as bone tumors, osteoporosis, and rheumatoid arthritis [52]. Zhao et al. [53] showed that *MEG3* could inhibit the osteogenic differentiation of human dental pulp stem cells via the *miR-543*/Smad ubiquitin regulatory factor 1/Runx2 axis. Similarly, downregulation of *MEG3* suppresses the osteogenic differentiation of PDLSCs through the *miR-27a-3p*/insulin-like growth factor (IGF) 1 axis in periodontitis [54]. In one study, researchers showed that the upregulation of *MEG3* suppresses osteogenic differentiation by downregulating BMP-2 expression in PDLSCs [55]. Furthermore, Chen et al. suggested that *MEG3*-mediated activation of BMP-4 signaling may promote the osteogenic differentiation of BMSCs. This process is regulated by the DEP domain-containing mammalian target of rapamycin-interacting protein.

### *GAS5*

In recent studies, many diseases have been shown to be associated with *GAS5* [1]. However, few studies have described the roles of *GAS5* in bone diseases. Feng et al. [1] showed that *GAS5* overexpression prevents the development of osteoporosis by promoting the osteogenic differentiation of MSCs via targeting *miR-498* to regulate Runx2. As a direct target of phosphatase and tensin homolog, *miR-26-5p* was shown to bind to *GAS5* [56]. *GAS5* can also promote osteogenic differentiation via the *miR-135a-5p*/FOXO1, growth differentiation factor 5, and p38/c-Jun N-terminal kinase signaling pathways [57, 58].

## Roles of the lncRNA *H19* in osteogenic differentiation

The lncRNA *H19* is transcribed from the *H19/IGF2* gene located on human chromosome 11p15.5 and has a molecular weight of 2.3 kilobase [59, 60]. Several studies have shown that *H19* is related to the development of cancer [61–64], and the *H19* locus can show tumor-suppressive effects in some cancers [65]. However, in oral squamous cell carcinoma, hepatocellular carcinoma, breast cancer, and bladder cancer, *H19* is aberrantly upregulated and can act as a biomarker [63].

*H19* is upregulated during the osteogenic induction of primitive stem cells and plays important functional roles in regulating osteogenic differentiation. The expression of *H19* varies during different stages of osteogenic differentiation. In some in vitro studies, the osteogenic differentiation of human adipogenic stem cells (hASCs) is induced by the inhibition of *H19* expression, resulting in the upregulation of the expression of pro-osteogenic genes. Additionally, overexpression of *H19* downregulates the expression of pro-osteogenic genes [66]. Liao et al. [67] firstly reported a method for the generation of functional *H19* using the AdEasy system and identified the biphasic effects of *H19* on MSC osteogenic differentiation in immortalized mouse adipose-derived progenitors.

**Fig. 2:**
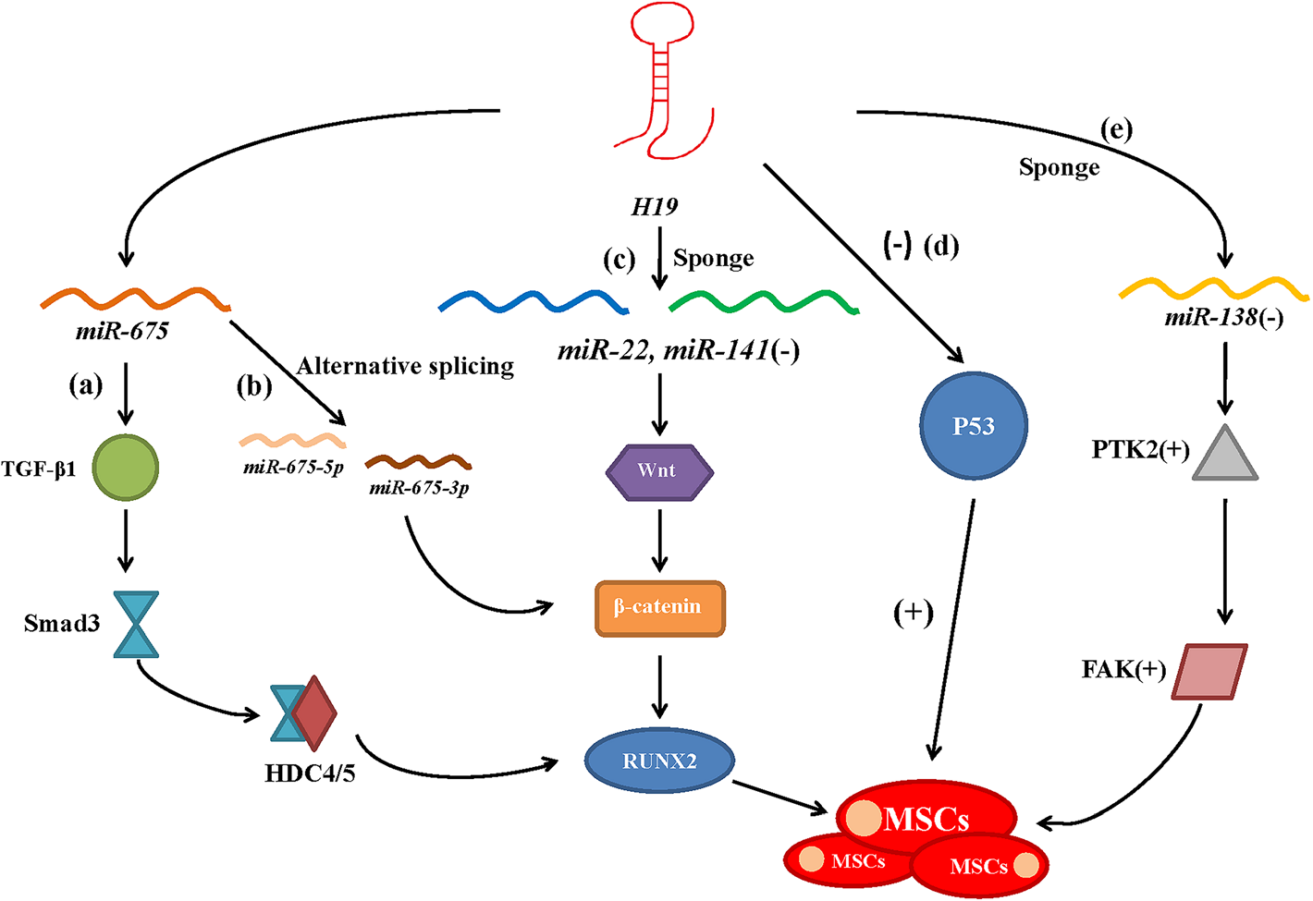
Roles of the lncRNA *H19* in osteogenic differentiation. *H19* regulates the osteogenesis of MSCs through different regulatory mechanisms, including classical mechanisms and signal pathways in the presence or absence of external stimuli. (**a**) *H19* downregulates TGF-β1 through *miR-675* and inhibits the phosphorylation of Smad3, suppressing the targeting of HDAC4/5 to the promoter of Runx2. (**b**) H19, as a precursor of *miR-675*, produces two mature miRNAs (*miR-675-5p* and *miR-675-3p*), which regulate osteogenic differentiation through the Wnt/β-catenin signaling pathway. (**c**) *H19* sponges with *miR-22* and *miR-141* to promote Wnt/β-catenin/Runx2 expression, thereby enhancing the osteogenic differentiation of MSCs. (**d**) *H19* binds directly to p53 protein, inhibits the activity of downstream targets of p53, and promotes the proliferation of MSCs. (**e**) The *miR-138* sponge, through competitive binding with *H19*, reduces the inhibition of the *PTK2* gene to promote FAK expression and induce the osteogenic differentiation of MSCs

Functionally, *H19* can participate in the regulation of osteogenic differentiation as an miRNA precursor. Moreover, *H19* can act as a competitive endogenous RNA by adsorbing and inhibiting the expression of miRNAs. Inhibition of *miR-22* and *miR-141* by *H19* results in the upregulation of Wnt/β-catenin/Runx2, thereby promoting the osteogenic differentiation of MSCs. The *miR-138* sponge, through competitive binding with *H19*, reduces the inhibition of *PTK2* gene expression to promote FAK expression and induce the osteogenic differentiation of MSCs [68]. Similarly, *H19* mediates ligand-dependent nuclear receptor corepressor to affect the osteogenic and adipogenic differentiation of BMSCs through sponging *miR-188* [69]. Additionally, *H19* also regulates osteogenic differentiation through various other signaling pathways. The *TP53* gene blocks cell cycle progression and inhibits cell proliferation by enhancing the transcription of different genes. During the osteogenic differentiation of MSCs, *H19* binds directly to the p53 protein, inhibits the activity of p53, and promotes the proliferation of osteoblasts from MSCs [70, 71]. In a C57/BL6 mouse strain and A2lox-miR-675 cells, Keniry et al. [60] showed that *H19* downregulates transforming growth factor (TGF)-1 expression through *miR-675*/TGF-1, inhibits the phosphorylation of Smad3, and downregulates histone deacetylase (HDAC) 4/5, enabling HDACs to target the promoter of *Runx2*. Other studies have also shown that *H19* can act as a precursor of *miR-675* and produce two mature miRNAs (*miR-675-5p* and *miR-675-3p*) by shearing, thereby regulating osteogenic differentiation through the Wnt/β-catenin signaling pathway [2, 72] (Fig. 2).

## Conclusions

Compared with coding RNAs and miRNAs, many lncRNAs have still not been extensively studied, and the mechanisms and functions of these lncRNAs have not been clarified. Importantly, various lncRNAs have been shown to play roles in bone regeneration and osteogenic differentiation. Additionally, advancements in technology have facilitated the study of lncRNAs in different fields. For example, RNA-binding protein immunoprecipitation (RIP) has been widely used to explore the interactions between proteins and lncRNAs in vivo. Then, after confirming the target protein, quantitative reverse transcription polymerase chain reaction can be used to isolate and quantify the lncRNA [73]. Wang et al. [74] used RIP to identify the association between the lncRNA *MIAT* and *miR-200a* in the differentiation of bone marrow-derived MSCs into endothelial cells in a rat model of erectile dysfunction [75]. Although the interactions of RNA-binding proteins with different RNAs are critical for RNA regulation, these interactions are difficult to detect. Crosslinking immunoprecipitation (CLIP) can also be used to solve this problem of identifying RNA/protein interactions in vivo [76, 77]. In CLIP, cells are irradiated with ultraviolet light to generate covalent bonds between the target RNA and protein when RNA/protein complexes come in close contact. After this step, RNA-binding proteins can be purified [75]. Moreover, RNA-pulldown assays and chromatin isolation by RNA purification can also be used to evaluate, identify, and test lncRNAs. However, the differential expression of many lncRNAs in various disease states and cell types has still not been clarified. Accordingly, bioinformatics studies, such as microarray analyses, are expected to have important applications in functional studies of lncRNAs. For example, Wang et al. [78] explored the potential roles of lncRNAs in ONFH via microarray and bioinformatics analyses of the lncRNA expression profiles of BMSCs isolated from patients with steroid-induced ONFH.

Overall, in this review, we summarized the functions and mechanisms of *H19*, which plays important roles in osteogenic differentiation. Many studies of *H19* regulation have been reported, including the regulatory effects of *H19* on gene expression, signaling pathways, lncRNA/miRNA sponging, and miRNA precursors. These mechanisms and potential biomarkers are expected to guide diagnoses, clinical treatments, and prognostic judgments in the future. However, the regulatory mechanisms of *H19* have not been fully elucidated. For example, there is still a lack of information regarding the microarray expression profiles of *H19-*overexpressing or *H19*-knockdown cells during osteogenic differentiation; thus, the effects of *H19* on the expression of downstream factors has not been determined. Such studies may improve our understanding of this important lncRNA. Studies of *H19* are still in the primary research stage, and the potential clinical applications of this lncRNA are unclear. However, the biological potential of lncRNAs is obvious, and further studies of the clinical importance of lncRNAs, including *H19*, in bone diseases, such as osteoporosis, fracture, and other diseases, may lead to improvements in therapeutic strategies for, and outcomes of, bone-associated diseases.

## Data Availability

Not applicable.

## Abbreviations

MSC: Mesenchymal stem cell
lncRNA: Long noncoding RNA
RNA seq: RNA sequencing
ncRNA: Noncoding RNA
siRNA: Small interfering RNA
miRNA: MicroRNA
CC: Cervical cancer
*DANCR*: Differentiation antagonizing non-protein-coding RNA
PDLSC: Periodontal ligament stem cell
Runx2: Runt-related transcription factor 2
BMP-2: Bone morphogenetic protein 2
*HOTAIR*: HOX transcript antisense RNA
ONFH: Nontraumatic osteonecrosis of the femoral head
*MALAT1*: Metastasis-associated lung adenocarcinoma transcript 1
Osx: Osterix
*MEG3*: Maternally expressed gene 3
CLIP: Crosslinking immunoprecipitation
RIP: RNA-binding protein immunoprecipitation
